# The Hyperpolarization-Activated Cyclic-Nucleotide-Gated Channel Blocker Ivabradine Does Not Prevent Arrhythmias in Catecholaminergic Polymorphic Ventricular Tachycardia

**DOI:** 10.3389/fphar.2019.01566

**Published:** 2020-01-17

**Authors:** Hanna Bueno-Levy, David Weisbrod, Dor Yadin, Shiraz Haron-Khun, Asher Peretz, Edith Hochhauser, Michael Arad, Bernard Attali

**Affiliations:** ^1^ Department of Physiology and Pharmacology, The Sackler Faculty of Medicine, Tel Aviv University, Tel Aviv, Israel; ^2^ Leviev Heart Center, Sheba Medical Center, Tel Aviv, Israel; ^3^ The Cardiac Research Laboratory, Felsenstein Medical Research Center, Rabin Medical Center, Tel Aviv University, Petah Tikva, Israel

**Keywords:** ivabradine, SK4, potassium channel, catecholaminergic polymorphic ventricular tachycardia, cardiac arrhythmia, ventricular arrhythmias, pacemaker

## Abstract

Catecholaminergic polymorphic ventricular tachycardia (CPVT) is an inherited, stressed-provoked ventricular arrhythmia. CPVT is treated by β-adrenergic receptor blockers, Na^+^ channel inhibitors, sympathetic denervation, or by implanting a defibrillator. We showed recently that blockers of SK4 Ca^2+^-activated K^+^ channels depolarize the maximal diastolic potential, reduce the heart rate, and attenuate ventricular arrhythmias in CPVT. The aim of the present study was to examine whether the pacemaker channel inhibitor, ivabradine could demonstrate anti-arrhythmic properties in CPVT like other bradycardic agents used in this disease and to compare them with those of the SK4 channel blocker, TRAM-34. The effects of ivabradine were examined on the arrhythmic beating of human induced pluripotent stem cells derived cardiomyocytes (hiPSC-CMs) from CPVT patients, on sinoatrial node (SAN) calcium transients, and on ECG measurements obtained from transgenic mice model of CPVT. Ivabradine did neither prevent the arrhythmic pacing of hiPSC-CMs derived from CPVT patients, nor preclude the aberrant SAN calcium transients. In contrast to TRAM-34, ivabradine was unable to reduce *in vivo* the ventricular premature complexes and ventricular tachyarrhythmias in transgenic CPVT mice. In conclusion, ivabradine does not exhibit anti-arrhythmic properties in CPVT, which indicates that this blocker cannot be used as a plausible treatment for CPVT ventricular arrhythmias.

## Introduction

Catecholaminergic polymorphic ventricular tachycardia (CPVT) is a rare, potentially fatal, inherited arrhythmia disease. It is often triggered by stress leading to polymorphic ventricular tachycardia in otherwise structurally normal hearts (Priori and Chen, 2011; Lieve et al., 2016). The pathophysiological mechanism of this disorder is suggested to involve diastolic Ca^2+^ leakage from the sarcoplasmic reticulum (SR), thereby producing local increase in cytosolic Ca^2+^ that is extruded by the Na^+^–Ca^2+^ exchanger NCX1. The increased NCX1 depolarizing activity generates early- or delayed-afterdepolarizations (EADs or DADs) that trigger premature beats and fatal polymorphic ventricular tachycardia (Priori and Chen, 2011). CPVT is a heterogeneous genetic disease, including autosomal dominant mutations in ryanodine receptor type 2 (RyR2, CPVT1), autosomal recessive mutations in calsequestrin 2 (CASQ2, CPVT2), and more rarely mutations in triadin or calmodulin. While CASQ2 mutants are “loss of function” mutations, the RyR2 mutations are “gain of function” mutations, both of which lead to diastolic Ca^2+^ leakage (Priori et al., 2002; Chopra and Knollmann, 2011; Priori and Chen, 2011; Arad et al., 2012; Lieve et al., 2016; Roston et al., 2017).

In addition to β-adrenergic receptor blockers, CPVT treatment includes inhibitors of Na^+^ channels such as flecainide, implantable defibrillator, and sympathetic denervation (Priori et al., 2002; Hayashi et al., 2009; Lieve et al., 2016; Roston et al., 2017). We have recently showed that SK4 K^+^ channels could be an additional therapeutic target for ventricular arrhythmias in CPVT (Haron-Khun et al., 2017). Recently, we found that TRAM-34, a selective blocker of SK4 K^+^ channels, decreased the β-adrenergic-triggered DADs and arrhythmic Ca^2+^ transients in human induced pluripotent stem cells derived cardiomyocytes (hiPSC-CMs) of CPVT2 patients bearing a mutation in calsequestrin 2 (CASQ2-D307H) and in sinoatrial node (SAN) from CASQ2-D307H knock-in (CASQ2 KI) mice (Haron-Khun et al., 2017). *In vivo* telemetric electrocardiograms (ECG) measurements showed that treatment with the SK4 channel blockers, TRAM-34 and clotrimazole, triggered sinus bradycardia and greatly reduced the ventricular arrhythmias of CASQ2-D307H KI and CASQ2 knockout mice at rest and following exercise (Haron-Khun et al., 2017).

Recently, the pacemaker channel inhibitor ivabradine was found to decrease digitalis-induced ventricular arrhythmias and short QT-induced arrhythmic features in Langendorff-perfused rabbit heart preparations (Frommeyer et al., 2017a; Frommeyer et al., 2017b). Ivabradine is usually prescribed to treat stable angina pectoris and in association with β-blockers to cure heart failure with left ventricular systolic dysfunction (Koruth et al., 2017). Ivabradine inhibits the pacemaker or funny current (If) in the SAN tissue, which is conveyed by hyperpolarization-activated, cyclic nucleotide-gated cation 4 (HCN4) channels and results in a decrease in the rate of diastolic depolarization and, consequently, the heart rate. Ivabradine also inhibits the human cardiac human ether-à-go-go related gene (hERG) K^+^ channel (IKr current), in a use-dependent manner, thereby prolonging the ventricular repolarization (Melgari et al., 2015). Overall, this causes an increase in the effective refractory period and in the post repolarization refractoriness, thus preventing premature excitations, which are usually a prelude of ventricular tachycardia.

The aim of the present work was to examine whether ivabradine could decrease the occurrence of arrhythmias found in cellular and animal model of CPVT. Results indicate that ivabradine was unsuccessful in reducing the occurrence of DADs in hiPSC-CMs derived from a CPVT2 patient. Similarly, ivabradine was unable to prevent the aberrant Ca^2+^ transients found in SAN and to reduce the ventricular arrhythmias observed in the ECG from CASQ2-D307H KI mice.

## Methods

### Drugs

Isoproterenol, clotrimazole, and ivabradine hydrochloride were purchased from Sigma, while TRAM-34 from Tocris. For *in-vivo* telemetric recordings, ivabradine-HCl was dissolved in saline while TRAM-34 was solubilized into peanut oil.

### Animals

SvEv mice (3–6 months old) homozygous for the CASQ2 D307H mutation (CASQ2 D307H KI) and matched wild-type (WT) mice were used in this study (Song et al., 2007). Mice were maintained and bred in a pathogen-free facility on regular rodent chow with free access to water and 12-h light and dark cycles. The procedures followed for experimentation and maintenance of the animals were approved by the Animal Research Ethics Committee of Tel Aviv University (M-14-063) in accordance with Israeli law and in accordance with the Guide for the Care and Use of Laboratory Animals (1996, National Academy of Sciences, Washington, DC).

### Human Induced-Pluripotent Stem Cell Culture and Cardiac Differentiation

Human induced pluripotent stem cells (hiPSC) derived from normal healthy individuals and from patients bearing the CASQ2 D307H mutation (CPVT2) were grown on mitomycin C-inactivated mouse embryonic fibroblasts (MEF), in order to maintain them in an undifferentiated state. Cells were maintained pluripotent in a culture medium containing 80% Dulbecco’s modified Eagle medium (DMEM) F-12 (Biological Industries), 20% Knock Out SR (Invitrogen), 2 mM L-glutamine, 0.1 mM β-mercaptoethanol (Gibco), and 1% nonessential amino acids (NEA) (Gibco), supplemented with 4 ng/ml basic fibroblast growth factor (bFGF) (Invitrogen). The medium was replaced daily until the colonies were ready to passage (every 4–5 days). For embryoid bodies (EBs) induction (d0), hiPSC colonies were removed from their MEF feeder by collagenase IV treatment and collected. After centrifugation, the cells were resuspended in EBs medium containing 80% DMEM (Gibco), 20% fetal bovine serum (FBS) (Biological Industries), 1% NEA, and 1 mM L-glutamine and plated on 58-mm Petri dishes. After 7 days of culture in suspension, EBs were plated on 0.1% gelatin-coated plates and checked daily until a spontaneous beating activity was visible. Because CASQ2 is lately expressed in hiPSC-CMs, 25 days-old EBs were used. The beating clusters were mechanically dissected from EBs, following a three-step dissociation protocol (Weisbrod et al., 2013). The hiPSC-CMs were isolated and plated on Matrigel-coated glass coverslips (13 mm diameter) in 24-well plates. The coverslips were then incubated at 37°C, and a recovery period of 2 days was given before any electrophysiological experiment was performed.

### Mouse Sinoatrial Node Dissection and Calcium Transient Measurements

WT and CASQ2 D307H KI mice were anesthetized with isoflurane and sacrificed by cervical dislocation. The heart was rapidly removed and transferred into Tyrode solution containing heparin. After the atria were pinned and the superior and inferior vena cava localized, the ventricles were removed. The SAN was anatomically identified between the superior and inferior vena cava, the crista terminalis, and the interatrial septum. SAN tissue preparations were dissected *ex vivo* from WT and CASQ2-D307H KI mice as previously described (Torrente et al., 2015). The dissected whole SAN tissue was pinned on a hand-made chamber and was incubated in a Tyrode solution containing 10 µM Fluo-4 AM (Thermo Fisher Scientific) and pluronic acid for 1 h at 37°C in the dark. The SAN tissue was washed in Tyrode at 37°C in the dark for 10 min before experiments. Fluorescence of calcium transients was recorded using a photomultiplier (PTi D-104) at 35°C and the analog signals were digitized using Digidata 1440 (Molecular Devices) and analyzed with pCLAMP 10.5 software.

### Electrophysiology

In all experiments, the coverslips were perfused at 33°C with an external solution containing (in mM): 140 NaCl, 4 KCl, 11 glucose, 1.2 MgCl2, 1.8 CaCl2, 5.5 4-(2-hydroxyethyl)-1-piperazineethanesulfonic acid (HEPES) titrated to pH 7.4 with NaOH and adjusted at 320 mOsm with sucrose. Whole-cell patch-clamp recordings were performed with an Axopatch 700B amplifier (Molecular Devices) and pCLAMP 10.5 software (Molecular Devices). Signals were digitized at 5 kHz and filtered at 2 kHz using microelectrodes with resistances of 4–7 MΩ were pulled from borosilicate glass capillaries (Harvard Apparatus) and filled with an intracellular solution containing (in mM): 130 KCl, 5 MgATP, 5 ethylene glycol-bis(β-aminoethyl ether)-*N*,*N*,*N*′,*N*′-tetraacetic acid (EGTA), 10 HEPES titrated to pH 7.3 with KOH and adjusted at 290 mOsm with sucrose. Unless otherwise stated, internal free calcium concentrations were 100 nM for current-clamp experiments and were titrated with EGTA and CaCl2 using the MaxChelator software (www.stanford.edu/~cpatton/maxc.html).

### *In Vivo* Telemetric Recordings

Telemetric ambulatory long-term ECG recordings, analogous to Holter monitoring in humans, were obtained with implantable transmitters. The investigator was blinded for the mice genotypes. WT, CASQ2-D307H KI SvEv mice were anesthetized with ketamine (75–90 mg/kg) and xylazine (5–8 mg/kg) intraperitoneally (IP) (Kepro, Holland), and a midline incision was made along the spine. An implantable 3.5 g wireless radiofrequency transmitter (DSI MM USA, device weight 3.8 g) was aseptically inserted into a subcutaneous tissue pocket in the back as previously described (Katz et al., 2010). Animals were allowed to recover after surgery at least 6 days before any experiments. Baseline ECG were obtained 15 min after IP injection of the appropriate vehicle (saline for ivabradine or peanut oil for TRAM-34). For pharmacological experiments, the same mouse was used a few hours after baseline ECG recordings (vehicle injection) and for subsequent ECG recordings upon IP injection of 6 mg/kg ivabradine or 20 mg/kg TRAM-34. Telemetered ECG tracings were obtained in conscious mice at rest for 1 min and during peak exercise (i.e. the first minute of recovery). In the treadmill exercise, mice were forced to exercise on a rodent treadmill; gradually increasing the speed up to a maximum of 15 m/min. Ventricular tachycardia (VT) was defined as four or more consecutive ventricular beats. If this phenotype was consecutively observed for more than 15 s, it was defined as “sustained” ventricular tachycardia (SVT). Shorter VTs were characterized as “non-sustained” (NSVT). All other ventricular arrhythmias, such as premature beats, ventricular bigeminy, couplets, and triplets were all defined as ventricular premature contractions (VPCs) (Katz et al., 2010).

### Data Analysis

Rate, DADs, and calcium transients were analyzed with the Clampfit program (pCLAMP 10.5; Molecular Devices). Sinus rhythm, PR interval, and ECG arrhythmic features were analyzed with the LabChart 8 Reader (ADInstruments). Data were analyzed with Excel (Microsoft) and Prism 5.0 (GraphPad Software) and are expressed as mean ± SEM. Statistical analysis was performed using the two-tailed paired Student t test and the linear regression for correlation or by one way ANOVA followed by Tukey’s or Bonferroni’s multiple comparison test. P values of less than 0.05 were assumed significant.

## Results

### Ivabradine Does Neither Affect the Firing Rate nor Prevent the Occurrence of Delayed-Afterdepolarizations in Human Induced Pluripotent Stem Cells Derived Cardiomyocytes Derived From CPVT2 (CASQ2-D307H) Patients

**Figure 1:**
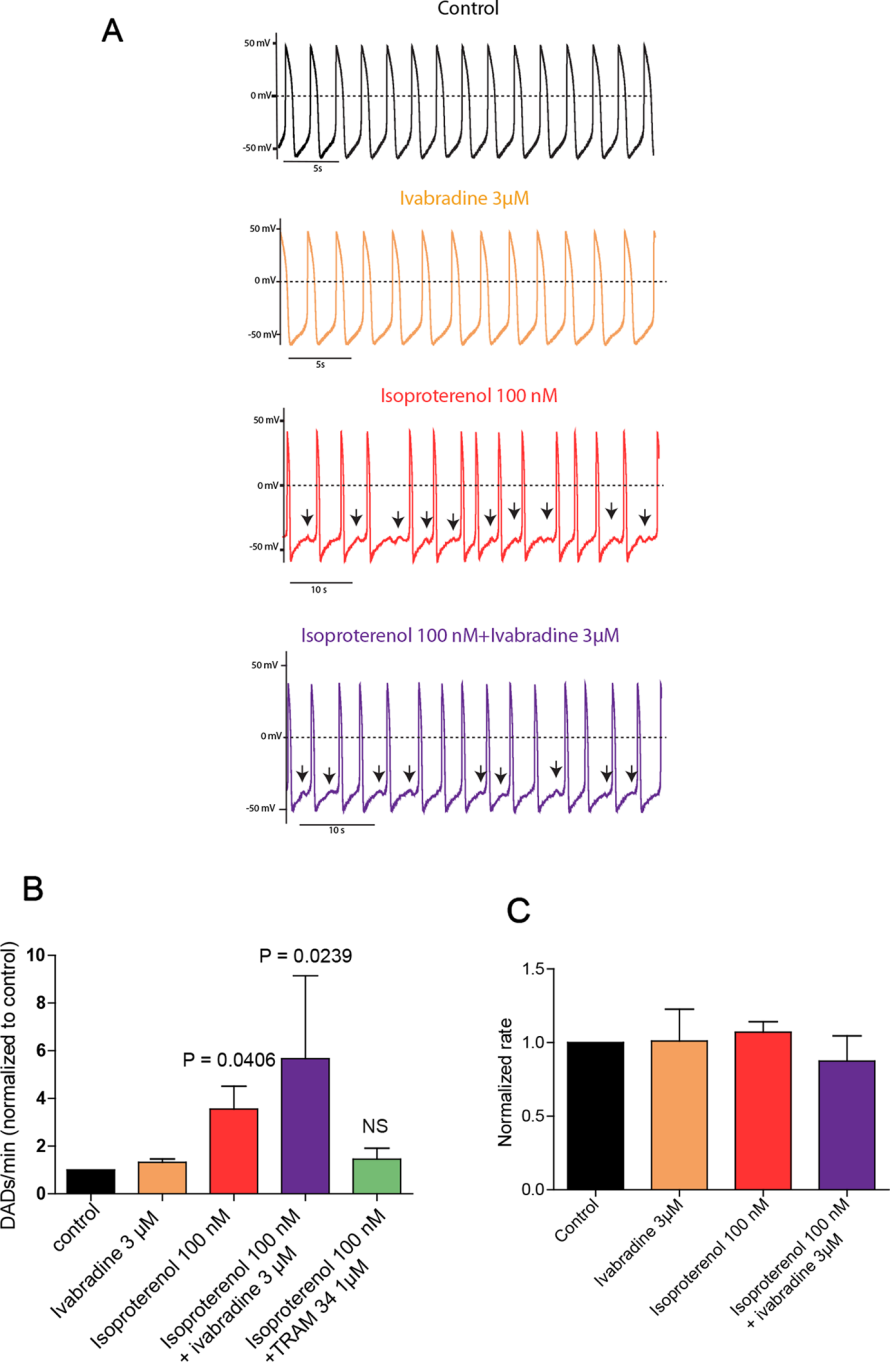
Ivabradine does not prevent the occurrence of delayed-afterdepolarizations (DADs) in human induced pluripotent stem cells derived cardiomyocytes (hiPSC-CMs) derived from CPVT2 (CASQ2-D307H) patients. **(A)** Representative traces of spontaneous action potential (AP) recorded in CASQ2 D307H hiPSC-CMs are shown from the same cell in the absence of any drug (black trace, control), then in the presence of 3 μM ivabradine (orange trace), 100 nM of isoproterenol (red trace), and both isoproterenol + ivabradine (purple trace). **(B)** Data summary for each treatment of the numbers of DADs, which were normalized to those obtained in the same cell before the addition of any drug. Isoproterenol (100 nM) increased DADs by 3.56 ± 0.96-fold [n = 19, P = 0.0147, one-way ANOVA (F = 3.402; P = 0.0147); Dunnett’s multiple comparisons test, P = 0.0406]; ivabradine (3 µM) + isoproterenol (100 nM) increased DADs by 5.67 ± 3.47-fold, n = 4, one-way ANOVA (F = 3.402; P = 0.0147); Dunnett’s multiple comparisons test, P = 0.0239; TRAM-34 (1 µM) + isoproterenol (100 nM) increased DADs by only 1.45 ± 0.41–fold, which is not significant (NS) compared to control; n = 15, one-way ANOVA (F = 3.402; P = 0.0147); Dunnett’s multiple comparisons test, P = 0.983. **(C)** Data summary of the normalized beating rates for each treatment. Beating rates were normalized those obtained in the same cell before the addition of any drug (control).The ANOVA test found no significant differences.

Isolated spontaneously beating hiPSC-CMs (25 days-old EBs) derived from normal (healthy) and CPVT2 patients carrying the CASQ2 D307H mutation (Novak et al., 2012) were used and their spontaneous firing properties were examined as previously shown (Haron-Khun et al., 2017). We recorded spontaneous action potentials fired from single hiPSC-CM cells in the current-clamp mode of the patch-clamp technique. Shown are representative traces of a spontaneously beating CPVT2-derived hiPSC-CM cell, which indicates that the application of 3 µM of ivabradine alone did not affect the beating rate (**Figures 1A, C**). In line with our previous findings (Haron-Khun et al., 2017), 100 nM isoproterenol did not increase the beating rate of CPVT2 hiPSC-CMs, in contrast to that of control healthy patients, but instead triggered DADs (**Figures 1A–C**). The number of DADs for each treatment was normalized to that obtained in the same cell before the addition of any drug. The records revealed a significant increase in the normalized number of DADs in the presence of 100 nM isoproterenol *versus* control [3.56 ± 0.96-fold, n = 19, one-way ANOVA (F = 3.402; P = 0.0147); Dunnett’s multiple comparisons test, P = 0.0406]. When 3 µM ivabradine was added together with isoproterenol, no bradycardic effect was seen and yet a significant increase in the normalized number of DADs was observed when compared to control [5.67 ± 3.47-fold, n = 4, one-way ANOVA (F = 3.402; P = 0.0147); Dunnett’s multiple comparisons test, P = 0.0239] (**Figures 1A–C**). Very similar results were obtained with a rather selective blocker of the If current, ZD7288 (25 µM), which in the presence of isoproterenol, was unable to prevent the occurrence of DADs compared to control [3.58 ± 0.8-fold, n = 5, one way ANOVA (F = 7.14; P = 0.0029), Dunnett’s multiple comparison test, P = 0.0224]. In agreement with our previous data (Haron-Khun et al., 2017), when the SK4 K^+^ channel blocker TRAM-34 (1 µM) was added to isoproterenol, no significant increase in the normalized number of DADs was revealed when compared to control [1.45 ± 0.46–fold, n = 15, one-way ANOVA (F = 3.402; P = 0.0147); Dunnett’s multiple comparisons test, P = 0.983] (**Figure 1B**). Taken together, our data show that in contrast to SK4 channel blockade, ivabradine, which inhibits I_f_ and hERG currents does not prevent the occurrence of arrhythmic features like DADs that accompany the spontaneous beating of CASQ2-D307H hiPSC-CMs in the presence of isoproterenol.

### Ivabradine Does Not Reduce the Arrhythmic Phenotype of Calcium Transients in Sinoatrial Node From CASQ2-D307H KI Mice

**Figure 2:**
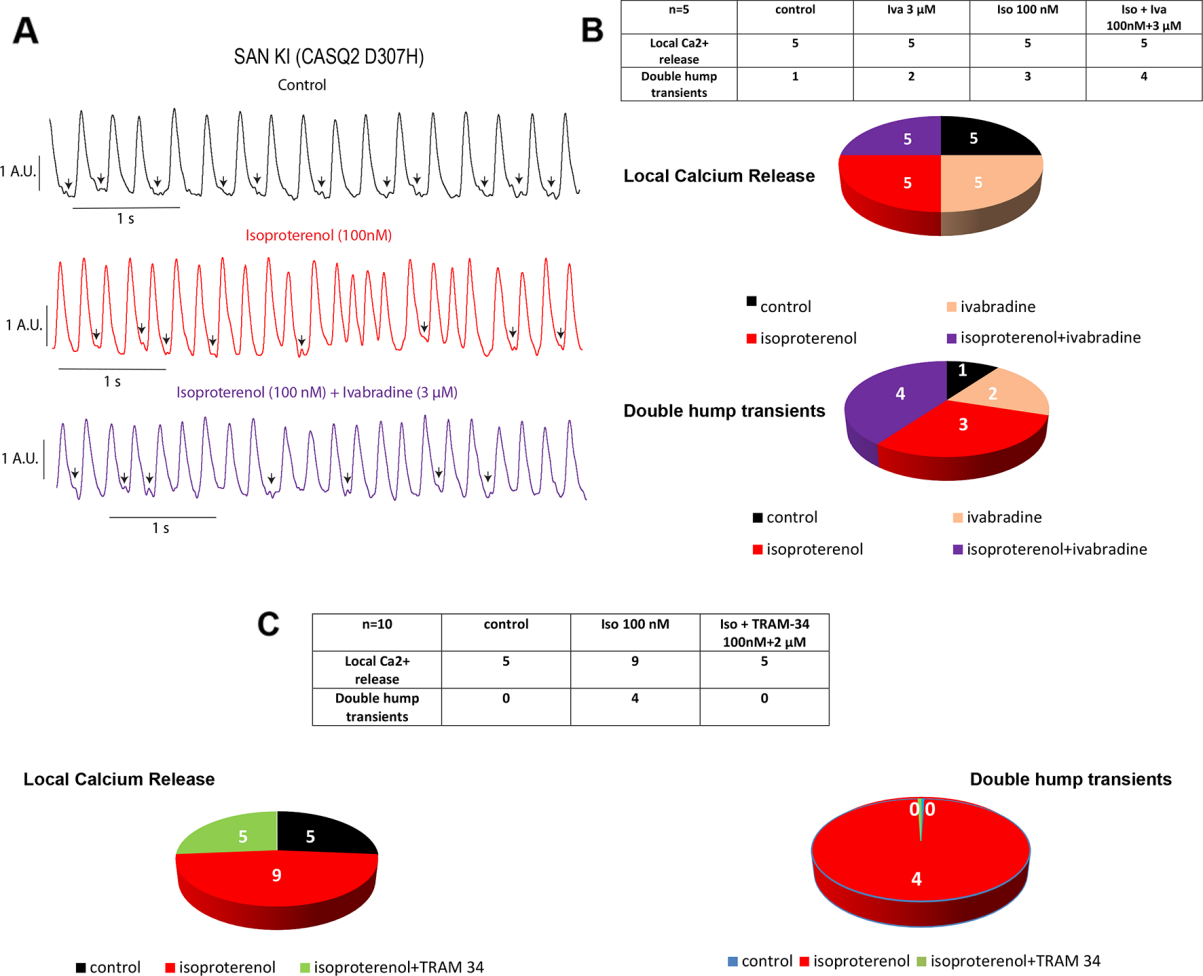
Ivabradine does not reduce the arrhythmic phenotype of calcium transients in sinoatrial node (SAN) from CASQ2-D307H KI mice. **(A)** Representative traces of calcium transients obtained from the same SAN of knock-in (KI) CASQ2 D307H mice under control (black trace), 100 nM isoproterenol (red trace), and isoproterenol + ivabradine (purple trace). **(B)** Data summary of the aberrant calcium transients observed in SAN from KI CASQ2 D307H mice under the described conditions (n = 5). **(C)** Data summary of the aberrant calcium transients observed in SAN from KI CASQ2 D307H mice under the described conditions (n = 10).

The spontaneous calcium transients of the SAN were investigated by exposing intact SAN tissue preparations dissected *ex vivo* from WT and CASQ2-D307H KI mice to Fluo-4 AM, as previously described (Torrente et al., 2015; Haron-Khun et al., 2017). We formerly showed that in SAN from WT mice, the rate of calcium transients was significantly larger in presence of 100 nM isoproterenol (Haron-Khun et al., 2017). Here, the exposure of WT SAN to 100 nM isoproterenol increased the rate of Ca^2+^ transients (from 2.9 ± 0.4 to 4.5 ± 0.3 Hz, n = 5, P = 0.015, one-way ANOVA and Tukey’s multiple comparison test). In contrast, the calcium transient rate of SANs from CASQ2-D307H KI mice was not significantly affected by the exposure to isoproterenol and/or ivabradine (**Figure 2A**). Importantly, the pattern of the calcium transients of SANs from CASQ2-D307H KI mice showed different abnormal features such as “local calcium release” (**Figure 2A**, arrows) and “double humps transients” both in control and isoproterenol conditions (**Figures 2A, B**). However, application of 3 µM ivabradine either alone or in the presence of 100 nM isoproterenol did not prevent the occurrence of “local calcium release” and “double humps transients” (**Figures 2A, B**). In line with our previous data (Haron-Khun et al., 2017), the SK4 channel blocker TRAM-34 (2 µM) behaved differently by decreasing the arrhythmic Ca^2+^ transient features events in the presence of isoproterenol (**Figure 2C**).

### Ivabradine Does Not Decrease *In Vivo* the Ventricular Arrhythmias Recorded in Electrocardiogram From CASQ2-D307H KI Mice

**Figure 3:**
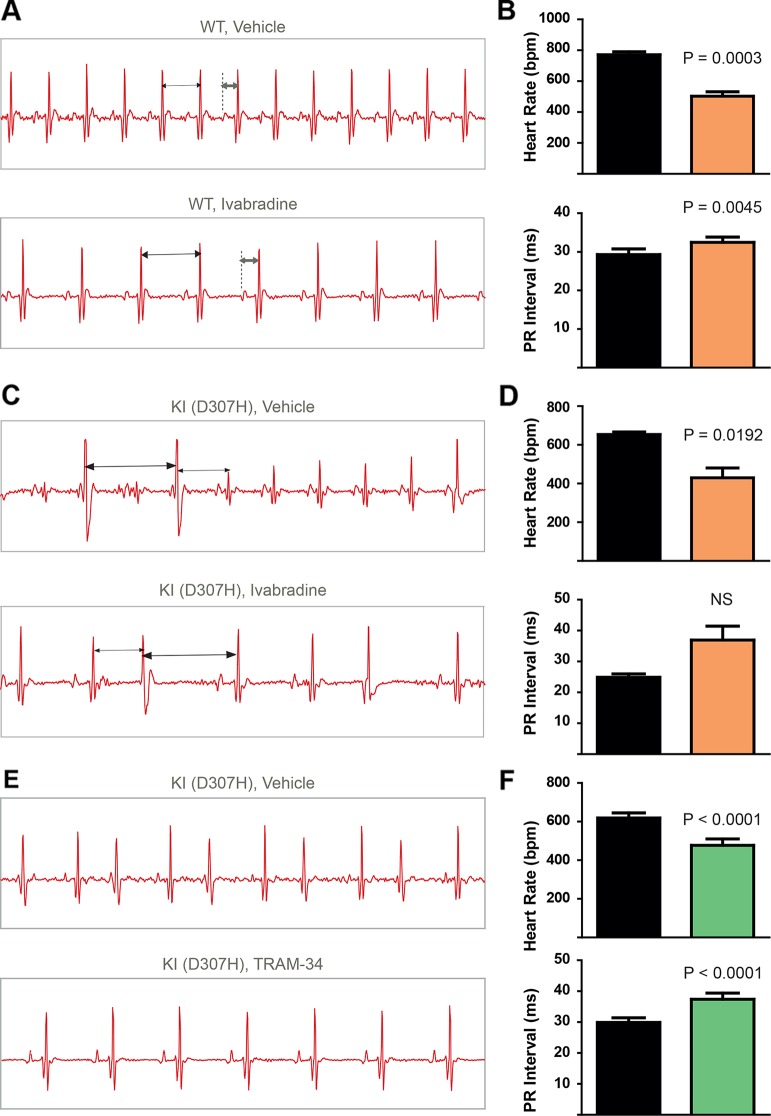
In contrast to TRAM-34, ivabradine does not prevent the ventricular arrhythmias recorded in ECG from CASQ2-D307H KI mice at rest. **(A)** Representative ECG recording following intraperitoneal (IP) injection of vehicle (saline, upper) and 6 mg/kg ivabradine (lower), in WT mice at rest. Sequential vehicle and ivabradine injections were performed on the same animal. **(B)** Data summary of heart rate (paired t test, P = 0.0003, n = 4) and PR interval (paired t test, P = 0.0045, n = 4) after IP injection of ivabradine. **(C)** Representative ECG recordings following IP injection of vehicle (saline, upper) and 6 mg/kg ivabradine (lower) in CASQ2 D307H knock-in (KI) mice at rest. **(D)** Data summary of the heart rate (paired t test, P = 0.0192, n = 4) and PR interval [not significant (NS), n = 4] after IP injection of ivabradine. **(E)** Representative ECG recording following IP injection of vehicle (peanut oil, upper) and 20 mg/kg TRAM-34 (lower) in CASQ2 D307H KI mice at rest. **(F)** Data summary of heart rate (paired t-test; P < 0.0001, n = 12) and PR interval (paired t-test; P < 0.0001, n = 12) in CASQ2 D307H KI mice at rest.

**Figure 4:**
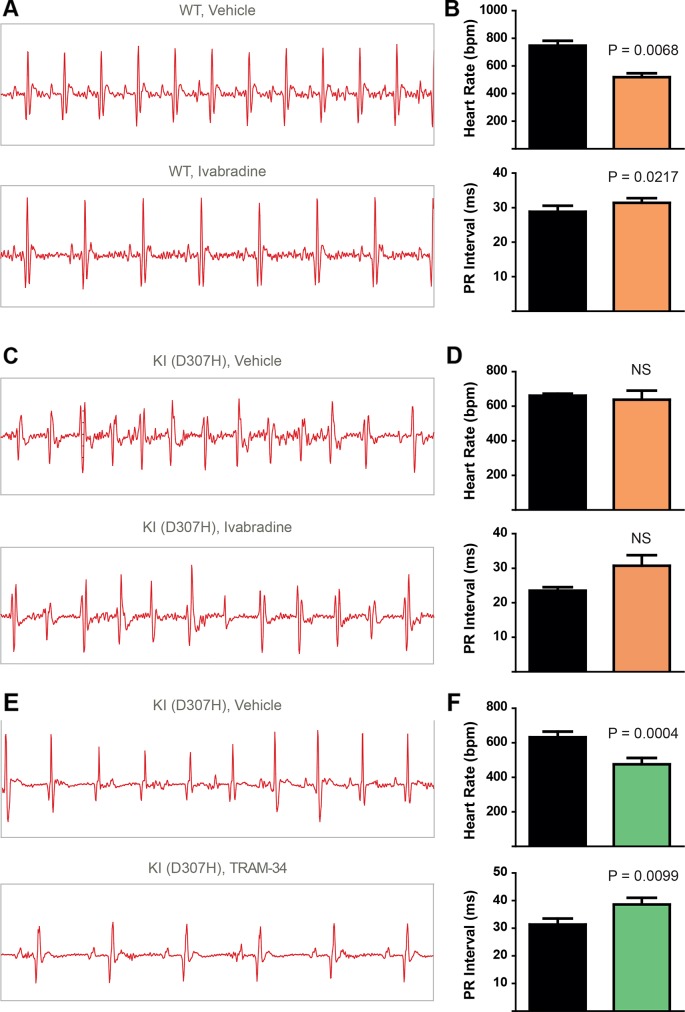
In contrast to TRAM-34, ivabradine does not prevent the ventricular arrhythmias recorded in ECG from CASQ2 D307H KI mice following treadmill exercise. **(A)** Representative ECG recording following intraperitoneal (IP) injection of vehicle (saline, upper) and 6 mg/kg ivabradine (lower), in WT mice following treadmill exercise. **(B)** Data summary of heart rate (paired t test, n = 4, P = 0.0068) and PR interval (paired t test, n = 4, P = 0.0217) in WT mice after treadmill exercise and IP injection of ivabradine. **(C)** Representative ECG recording following IP injection of vehicle (saline, upper) and 6 mg/kg ivabradine (lower) in CASQ2 D307H knock-in (KI) mice following treadmill exercise. **(D)** Data summary of heart rate [not significant (NS), n = 4] and PR interval [not significant (NS), n = 4] after IP injection of ivabradine. **(E)** Representative ECG recording following IP injection of vehicle (peanut oil, upper) and 20 mg/kg TRAM-34 (lower) in CASQ2 D307H KI mice following treadmill exercise. **(F)** Data summary of heart rate (paired t-test; P = 0.0004, n = 11) and PR interval (paired t-test; P = 0.0099, n = 11) in CASQ2 D307H KI mice following treadmill exercise.

To record continuously ECGs at rest and during treadmill exercise, a heart telemetry device was implanted in WT and CASQ2-D307H KI mice. For each session, continuous ECG recording was performed with the same animals receiving first IP injection of vehicle (saline or peanut oil) and then the blocker. In WT mice, ivabradine (6 mg/kg) exhibited, as expected, a bradycardic effect at rest, thereby decreasing the heart rhythm (from 771 ± 19 to 503 ± 26 beats/min for saline and ivabradine injection, respectively; n = 4, paired t-test P = 0.0003) and increasing the PR interval (from 29 ± 1 to 32 ± 1 ms, for saline and ivabradine injection, respectively; n = 4, paired t-test P = 0.0045) (**Figures 3A, B**, see arrows). Ivabradine produced similar bradycardic effects on WT mice during treadmill exercise (heart rate of 747 ± 35 and 518 ± 28 beats/min, for saline and ivabradine injection, respectively; n = 4, paired t-test P = 0.0068 and PR interval of 29 ± 2 *versus* 31 ± 1 ms for saline and ivabradine injection, respectively, n = 4, paired t-test P = 0.0217) (**Figures 4A, B**). In CASQ2-D307H KI mice, ivabradine (6 mg/kg) still produced bradycardic effect at rest (from 652 ± 18 to 444 ± 68 beats per min, for saline and ivabradine injection, respectively n = 4, paired t-test P = 0.0192), but not following treadmill exercise. Ivabradine poorly prevented the ECG arrhythmic features of CASQ2-D307H KI mice, where at rest three out of six mice still exhibited ventricular tachycardia or premature ventricular complexes (**Figures 3C, D**, **Table 1**). During treadmill exercise, ivabradine was completely ineffective since all six ivabradine-treated CASQ2-D307H KI mice showed ventricular tachycardia (**Figures 4C, D**, **Table 1**). In contrast, 9 out of 12 TRAM-34-treated (20 mg/kg) CASQ2-D307H KI mice exhibited normal sinus rhythm at rest, while following treadmill exercise, 4 out of 12 animals remained with normal sinus rhythm without significant ventricular arrhythmia (**Figures 3E, F** and **Figures 4E, F** and **Table 1**). These results show that ivabradine is unable to decrease the ventricular arrhythmias observed in ECG of CASQ2-D307H KI mice, as opposed to TRAM-34, which greatly reduces them (Haron-Khun et al., 2017).

**Table 1:** Comparative effects of ivabradine and TRAM-34 on the arrhythmogenic features of CPVT2 CASQ2-D307H knock-in (KI) mice at rest or during treadmill exercise.

Number of mice (n)	Vehicle 6	Ivabradine (6 mg/kg) 6	Vehicle 12	TRAM-34 (20 mg/kg) 12
KI at rest				
Normal sinus rhythm	0	3	3	9
VPC	2	1	6	3
NSVT	0	0	3	0
SVT	4	2	0	0
KI treadmill exercise				
Normal sinus rhythm	0	0	1	4
VPC	1	0	2	4
NSVT	0	0	9	4
SVT	5	6	0	0

The data show the comparative effects of ivabradine and TRAM-34 on the arrhythmogenic features of CPVT2 CASQ2-D307H KI mice at rest or during treadmill exercise. Ivabradine (6 mg/kg) was dissolved in saline, while TRAM-34 (20 mg/kg) was prepared in peanut oil and were injected intraperitoneally into 100 µl vehicle. VPC, ventricular premature complexes; NSVT, non-sustained ventricular tachycardia; SVT, sustained ventricular tachycardia. The types of arrhythmic features were classified following their severity: sinusal rhythm (normal), ventricular premature contractions (VPC), NSVT, and SVT.

## Discussion

In a recent study, we showed that SK4 channel blockers decrease the heart rate, depolarize the maximal diastolic potential (MDP), and attenuate ventricular arrhythmias in transgenic CPVT mice. In this work, we examined both *in vitro* and *in vivo* whether ivabradine could exhibit anti-arrhythmic properties in CPVT as other bradycardic agents such as β-adrenergic receptor blockers or SK4 channel inhibitors. Ivabradine is a blocker of the hyperpolarization-activated cyclic-nucleotide gated-channel, which mediates the cation non-selective “funny” current If, and is mainly encoded by the HCN4 protein in the sinoatrial node (Bucchi et al., 2013). In the SAN tissue, the If current is responsible for the cardiac pacemaker activity by generating the diastolic depolarization. Ivabradine blocks the HCN4 inner pore leading to a reduction in the slope of the diastolic depolarization, thereby slowing the heart rate (Bucchi et al., 2013). Ivabradine blocks the HCN channel in its open state and exhibits a use-dependence effect, becoming more potent at faster heart rates. As a bradycardic agent, ivabradine has been evaluated and is currently used in selected patients with systolic heart failure and stable angina (Koruth et al., 2017). Indeed, various experimental and epidemiological studies have found that atherosclerosis, heart failure, coronary artery disease, stroke, and arrhythmias are linked to elevated heart rate (Koruth et al., 2017). In addition to blocking HCN channels, studies showed that ivabradine prolonged ventricular action potentials by blocking the IKr current (hERG) in ventricular myocytes (Melgari et al., 2015). Moreover, it was shown that ivabradine blocks the recombinant hERG current over a range of concentrations overlapping with those required to block HCN4 (Melgari et al., 2015). Because of its concurrent inhibitory action on If and IKr currents, ivabradine was suggested to be a potential antiarrhythmic drug thanks to an increase in both the effective refractory period and the post-repolarization refractoriness (Frommeyer et al., 2017a; Frommeyer et al., 2017b). The bradycardic features of ivabradine raise the question of whether it could be effective in ventricular arrhythmias. Recently, ivabradine was found to decrease digitalis-induced ventricular arrhythmias and short QT-induced arrhythmic features in Langendorff-perfused rabbit heart preparations (Frommeyer et al., 2017a; Frommeyer et al., 2017b). Along with this rationale, we asked in the present work whether ivabradine could prevent the ventricular arrhythmias found in cellular and animal models of CPVT2. We found that ivabradine did not prevent the occurrence of DADs in the presence of isoproterenol in hiPSC-CMs of CPVT2 patients (**Figure 1**). In contrast, the SK4 channel blocker TRAM-34, significantly reduced the number of DADs, in line with our recent data (Haron-Khun et al., 2017) (**Figure 1**). Similarly, we showed that ivabradine was ineffective in preventing the arrhythmic calcium transients recorded in the presence of isoproterenol in SAN intact tissue from CASQ2 KI mice (**Figure 2**). However, TRAM-34 significantly decreased the occurrence of local Ca^2+^ release and double hump transient events in agreement with our previous results (Haron-Khun et al., 2017). Along the same line, ivabradine did not prevent the ventricular arrhythmias revealed by telemetry in CASQ2-D307H KI mice at rest and was totally ineffective during treadmill exercise (**Figures 3** and **4**, **Table 1**). A contrasting picture was obtained with TRAM-34, which significantly reduced the occurrence of ventricular arrhythmias at rest and to lesser extent upon treadmill exercise (**Figures 3** and **4**, **Table 1**).

We and others previously showed that CASQ2 knockout (CASQ2 KO) and CASQ2-D307H KI mice are significantly bradycardic (Glukhov et al., 2015; Haron-Khun et al., 2017). It is interesting to note that isoproterenol did increase neither the beating rate of hiPSC-CMs derived from CPVT2 patients nor the rate of Ca^2+^ transients of SAN taken from CASQ2 KI mice upon isoproterenol application. Similarly, in the ECG recording from CASQ2-D307H KI mice, the heart rate at rest was not increased following treadmill exercise (652 ± 18 and 658 ± 15 beats/min, respectively). A recent work (Ho et al., 2018) found that sympathetic stimulation failed to accelerate heart rate in exercised CASQ2 KO mice, suggesting a sympathetic-parasympathetic imbalance in CASQ2 KO and CASQ2-D307H KI mice, possibly due to autonomic adaptation.

Therapies for CPVT include exercise restriction, β-blockers, additional drugs, primarily flecainide, implantable cardioverter defibrillators (ICD) in high-risk patients to prevent sudden death, and sympathetic denervation (Priori et al., 2002; Hayashi et al., 2009; Lieve et al., 2016; Roston et al., 2017). Administration of β-blockers remains the longstanding cornerstone of treatment. However, the response to β-blockers is partial and often decreases with time because of an escape phenomenon (Priori et al., 2002; Hayashi et al., 2009). In addition, β-blockers exhibit undesirable side effects, including psychological depression, bronchospasm, peripheral vasoconstriction, hypotension, leg tiredness, and erectile dysfunction. Flecainide was recently shown to possess antiarrhythmic properties in CPVT through either a stabilizing effect on RyR2 by decreasing its opening probability or by increasing the threshold for triggered activity through its Na^+^ channel blocking activity (Lieve et al., 2016; Roston et al., 2017). However, routine use of flecainide monotherapy is not recommended yet because of lack of large retrospective studies. ICD implantation in young and active patients necessitates routine device replacement and is associated with device malfunction, including inappropriate shocks, infection, and psychological problems. The major clinical indications for left cardiac sympathetic denervation (LCSD) are ß-blocker intolerance or refractoriness, high risk of sudden death with ß-blocker treatment, or frequent ICD shocks (Lieve et al., 2016; Roston et al., 2017). However, LCSD is still rarely used as a supplementary therapy to ß-blocker administration and ICD implantation. Although tremendous progress has been made in the recent years in the treatment of CPVT, there is still a great need to develop additional or alternative new therapies. The gene therapy is of great potential in this context but is not yet ready for clinical use (Kurtzwald-Josefson et al., 2017).

A common feature of most pharmacological agents in use is their negative chronotropic effect. In this context, ivabradine treatment was a legitimate candidate drug to be tested in cellular and animal models of CPVT. Despite its bradycardic action, ivabradine was unable to prevent *in vitro* the occurrence of DADs and abnormal Ca^2+^ transients in the presence of isoproterenol as well as *in vivo* the ventricular arrhythmias in CASQ2-D307H KI mice. In contrast, we previously showed the SK4 channel blockers TRAM-34 and clotrimazole, though similarly reducing the heart rate and increasing the PR interval, could successfully reduce the occurrence of ventricular arrhythmias (Haron-Khun et al., 2017). This raises the question of why ivabradine and the SK4 channel blockers (TRAM-34 and clotrimazole) do not exert the same beneficial effect in CPVT ventricular arrhythmias, although both are bradycardic agents. First, it suggests that bradycardia and increase in the effective refractory period and in the post repolarization refractoriness are not sufficient to prevent ventricular arrhythmias in CPVT. Second, although the precise mechanism of action of SK4 K^+^ channels in the cardiac SAN cycle remains to be determined, normal SAN automaticity is regulated by coupling between an “M clock,” the ensemble of surface membrane ion channels, and a “Ca^2+^ clock,” the SR (Lakatta and DiFrancesco, 2009). According to the coupled-clock hypothesis, the M and Ca^2+^ clocks should crosstalk. Ca^2+^-calmodulin-activated adenylyl cyclase in pacemaker cells generates high cyclic AMP (cAMP) activity that activates the If channel by positively shifting the If channel activation. In addition, cAMP production controls protein kinase A (PKA)-dependent phosphorylation activity. PKA, together with Ca^2+^-activated calmodulin-dependent kinase II, phosphorylates various membrane clock channels (e.g., L-type Ca^2+^ channels and K^+^ channels), as well as Ca^2+^ clock proteins (Behar and Yaniv, 2016). Because of this coupling mechanism, it is possible that treatment with ivabradine alone is not sufficient to prevent ventricular arrhythmias in CPVT. Nevertheless, it was previously shown that the bradycardic effect of ivabradine was accompanied by reduced SR Ca^2+^ load, slowed intracellular Ca^2+^ cycling kinetics, and prolongation of the period of spontaneous local Ca^2+^ releases occurring during diastolic depolarization (Yaniv et al., 2013). It is possible that reduction in adenylate cyclase-cAMP/PKA activity (e.g., by β-blockers) in parallel to treatment with ivabradine could provide a more effective anti-arrhythmic therapy in CPVT. In line with this idea, a recent case report showed that ivabradine in combination with the β-blocker nadolol, successfully suppressed non-sustained polymorphic ventricular tachycardia in two patients heterozygous for RyR2 mutation (CPVT1) (Vaksmann and Klug, 2018). We recently suggested by a numerical model that SK4 channel contribution becomes significant only at the late repolarization, a crucial period for the Ca^2+^ clock and for the coupling to the voltage clock, thereby contributing to the MDP hyperpolarization, facilitating activation of If and recovery from inactivation of voltage-gated Ca^2+^ channels (Haron-Khun et al., 2017). Thus, inhibitors of SK4 Ca^2+^-activated K^+^ channels are ideally suited to suppress the coupling between the two clocks and their subsequent blockade may favorably act to prevent premature excitations, which are a prelude of ventricular arrhythmias.

In all, in contrast to SK4 channel blockers ivabradine does not exhibit anti-arrhythmic properties in CPVT and this drug alone cannot be used to treat CPVT ventricular arrhythmias.

## Data Availability Statement

All datasets generated for this study are included in the article.

## Ethics Statement

The animal study was reviewed and approved by the Animal Research Ethics Committee of Tel Aviv University (M-14-063) in accordance with Israeli law and in accordance with the Guide for the Care and Use of Laboratory Animals (1996, National Academy of Sciences, Washington, DC).

## Author Contributions

HB-L, DW, DY, SH-K, and AP performed the experiments and analyses. EH provided experimental tools. MA and BA conceived the project, planned the experiments, and wrote the manuscript.

## Conflict of Interest

The authors declare that the research was conducted in the absence of any commercial or financial relationships that could be construed as a potential conflict of interest.
